# Probing T Cell 3D Mechanosensing With Magnetically-Actuated Structures

**DOI:** 10.3389/fimmu.2021.704693

**Published:** 2021-08-09

**Authors:** Chirag Sachar, Lance C. Kam

**Affiliations:** Department of Biomedical Engineering, Columbia University, New York, NY, United States

**Keywords:** mechanobiology, CD4^+^ T cell, magnetic, micropillar, activation

## Abstract

The ability of cells to recognize and respond to the mechanical properties of their environment is of increasing importance in T cell physiology. However, initial studies in this direction focused on planar hydrogel and elastomer surfaces, presenting several challenges in interpretation including difficulties in separating mechanical stiffness from changes in chemistry needed to modulate this property. We introduce here the use of magnetic fields to change the structural rigidity of microscale elastomer pillars loaded with superparamagnetic nanoparticles, independent of substrate chemistry. This magnetic modulation of rigidity, embodied as the pillar spring constant, changed the interaction of mouse naïve CD4^+^ T cells from a contractile morphology to one involving deep embedding into the array. Furthermore, increasing spring constant was associated with higher IL-2 secretion, showing a functional impact on mechanosensing. The system introduced here thus separates local substrate stiffness and long-range structural rigidity, revealing new facets of T cell interaction with their environment.

## 1 Introduction

T cells are key agents of the adaptive immune response, coordinating precise and robust protection against pathogens, but in other settings also contributing to a range of diseases. These cells can also be leveraged to treat disease, and there is growing interest in the design of materials that direct ex vivo production of these living drugs in the context of cellular immunotherapy. In particular, tuning the mechanical properties of a biomaterial used to activate T cells can enhance subsequent function including cytokine secretion, proliferation, and population expansion (1–4). The early studies investigating T cell mechanosensing were carried out with flat, planar surfaces presenting ligands to the T Cell Receptor (TCR) and CD28, which provide activating and costimulatory signaling, respectively (3, 5). However, interactions between T cells and antigen presenting cells (APCs) are topographically complex, involving cellular protrusions, extensions, and other features that are defined over a range of spatial scales (6, 7). Subsequent studies using microstructured surfaces (8–11) showed that T cells can interact intimately with these topographies in model systems, altering a range of outputs associated with cell activation and function. Notably, T cells appear to respond to the structural rigidity of microscale elastomer pillars organized into long-range arrays, a system originally developed for measuring forces exerted by cells onto an underlying substrate (12). T cells respond to changes in pillar rigidity, expressed as that structure’s spring constant (the ratio of force applied tangentially at the tip to the tip deflection), by modulating microtubule organizing center (MTOC) transport and cytokine secretion (10). These experiments were carried out by altering the geometry (height and cross section) of each pillar, as previously described for other types of cells (13–15). This approach addressed a frequently-voiced critique of mechanobiology studies using planar substrates such as hydrogels in that stiffness was controlled through material chemistry; changing cross-linker density could affect local, nanoscale interactions with cells rather than substrate modulus alone (16). Microscale structuring allowed control over the larger-range mechanical response of a substrate while using the same material formulation, but introduced other issues. Changing parameters such as pillar width and depth also alters features such as local substrate curvature, area avaiable for cell adhesion, and/or nutrient availability which may alter cell response independently of system rigidity. This report introduces the use of magnetic fields to change the rigidity of micropillar arrays while keeping the structure dimensions constant, addressing such concerns.

**Figure 1:**
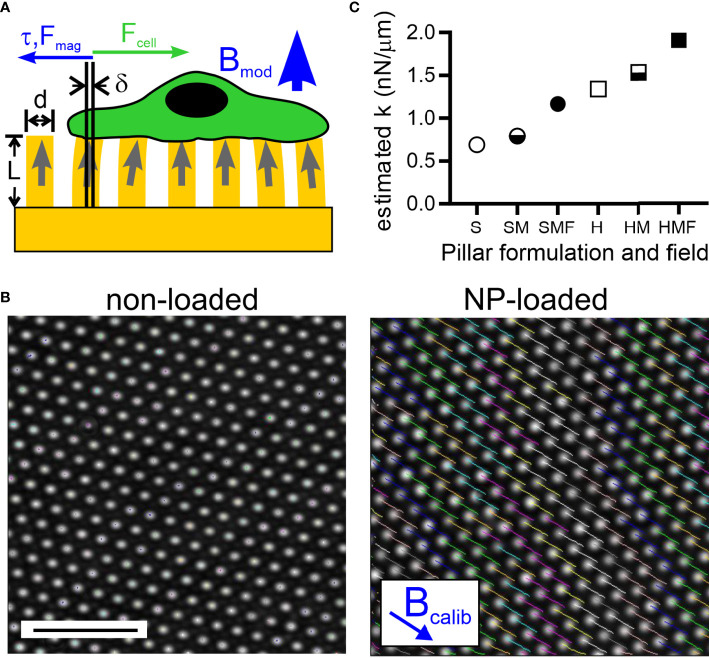
Magnetic modulation of pillar rigidity. **(A)** A magnetic field applied perpendicularly to an array of pillars (thus along the axis of individual pillars, as indicated by B_mod_) loaded with magnetic nanoparticles (NPs) produces a torque and associated force (τ, F_mag_) that counter deflections, such as those induced by an adherent cell (F_cell_). **(B)** Application of a magnetic field tangentially to the array (thus perpendicular to the axis of individual pillars, as indicated by B_calib_) causes deflection of pillars that are loaded with NP’s (right panel). Pillars without NPs do not respond to the applied field (left panel). Scale bar = 10 µm. **(C)** Estimation of the spring constant presented by individual pillars, modulated by controlling the bulk Young’s modulus of elastomer used to fabricate these arrays (Soft, S, or Hard, H), loading of pillars with NPs (M), and application of a magnetic field (F).

The approach introduced here (**Figure 1A**) is a variation of a magnetic actuation micropillar system introduced by Sniakecki et al. (17). In that system, magnetic wires are embedded inside individual pillars. Application of a magnetic field tangential to the arrays (along the direction of multiple pillars and thus perpendicular to an individual pillar) imparts a torque to the wire. Since the wire is anchored to the substrate through the pillar, this torque is transmitted to adherent cells as a lateral force. In the present study, the magnetic field is applied perpendicular to the arrays rather than tangential, in the direction represented by B_mod_ in **Figure 1A**. The pillars are loaded with superparamagnetic nanoparticles (NPs). A magnetic field applied in this configuration induces a moment along the pillar axis which will tend to align with the field. Deflection of the pillar away from this alignment, for example by a cell producing F_cell_ in **Figure 1A**, will produce a restoring torque, τ, that seeks to realign the magnetic moment and field. The magnitude of this torque and resultant force applied to the cell are approximately linear to pillar tip deflection for small displacements. In short, application of a magnetic field in this configuration increases the apparent spring constant of NP-loaded pillars. This approach promises the ability to change the structural rigidity of a substrate independent of geometry or material formulation. This approach also avoids the need to introduce chemical or biomolecular agents into the system. Here, we use this magnetic actuation platform to investigate the interaction of T cells with topographically complex surfaces.

## 2 Materials and Methods

### 2.1 Cell Culture

Mouse CD4+ T cells were isolated from the spleens of C57BL/6 mice, age 6-10 weeks. After filtering through a 40 μm mesh, naive CD4+ cells were enriched *via* negative selection using the Miltenyi CD4^+^ T cell isolation system. Complete culture media consisted of RPMI 1640 supplemented with 10% fetal bovine serum, 10 mM HEPES, 2 mM L-glutamine, 50 µM β-mercaptoethanol (Sigma), 50 U/mL penicillin, and 50 µg/mL streptomyosin, and 50 U/mL rhIL-2 (Peprotech), all reagents from Thermo unless otherwise noted. Cells were used immediately in experiments as described below. Incubations were carried out under standard cell culture conditions (37°C, 5% CO_2_/95% air). For live-cell experiments, this environment was maintained using a Tokai Hit stage top incubation system. For inhibition experiments, cells were pretreated with inhibitor in complete culture media for 15 minutes, and then seeded onto experimental surfaces. Inhibitors included Y-27632 (ROCK inhibitor, 20 μM; Sigma-Aldrich) and CK-666 (Arp2/3 inhibitor, 100 μM; Sigma-Aldrich).

### 2.2 Elastomer Pillar Fabrication

Arrays of elastic micropillars were fabricated following a double-casting approach described by Tan et al. (12). Briefly, masters of the microscale pillar arrays were generated by nanolithography as previously described (10, 11, 18). Each array is made up of roughly 1000 by 1000 pillars. Individual pillars are of 6 μm height and 1 µm diameter of 1 μm, spaced 2 µm center-to-center in hexagonal arrays. Negative molds, containing pits that are the negative of the pillars, were prepared by pouring polydimethylsiloxane (PDMS, Sylgard 184, Dow Corning, mixed at the manufacturer-specified elastomer base: cross-linker ratio of 10:1) onto the silicon masters then curing for 8 hours at 65°C. These negative molds were then silanized over night with (tridecafluoro-1,1,2,2,-tetrahydrooctyl)-1-trichlorosilane (United Chemical Technologies). The negative molds were used to cast PDMS pillar arrays (curing at 65°C for 8 hours) directly onto glass coverslips (thickness #0 Fisherbrand) (**Figure 3**). To release the pillars, the molds were inverted, peeled off in 100% ethanol to prevent pillar collapse, and the remaining upright pillars were washed in 3X phosphate-buffered saline (PBS).

The PDMS used to create the arrays consisted of either Sylgard 184 alone or Sylgard 527 mixed with Sylgard 184 at a ratio of 1:3 (v/v), each prepared following the manufacturers specifications (Dow Corning). The stiffness of each elastomer was estimated by indentation, using a flat cylindrical head of 8-mm diameter (D). Young’s modulus (E) was calculated from the deflection of the surface (h) in response to an applied weight of specified mass (m), using the Hertizan contact model, with Poisson ratio (ѵ) = 0.5 and gravitational field (g) = 9.81 m/sec^2^,

$$\text{E}=(1-v^{2})\text{m}(\frac{\text{g}}{\text{Dh}})$$

The samples used for these measurements were approximately 1 cm thick, and surface deflection was less than 10% of that depth.

### 2.3 Magnetic Pillar Fabrication

To fabricate magnetic pillars, the negative pillar molds were loaded with superparamagnetic nanoparticles (NP, 10-nm magnetite, EMG 1200) prior to casting of PDMS. The NP solution was prepared by dissolving 0.06 g of dry powder in 3mL Toluene in a 15mL round flask using a vortexer. Then, 5mL of Hexane and 5 mL of Hexadecane were added into the solution, and the solution was poured into a Teflon beaker. A drill mixer was used to dissolve the particles into the organic solvent for 5 minutes. Then, the mixture was sonicated with a wand sonicator (Branson SFX150 Sonifier) for 5 minutes. Finally, the mixture was sonicated in a bath (Branson B1510-MT) for 20 minutes. In preparation for loading, negative molds were placed on top of a neodymium permanent magnet (DXO8B, 1” D X 1/2” H cylindrical, N52, K&J magnetics). NPs were introduced into the negative molds using multiple loading cycles. For each cycle, 15 uL of the NP solution was placed directly onto the mold tops and allowed to sit for 15 minutes. The molds were then placed in a centrifuge dish, and 10 more microliters of the NP solution added to the system. Molds were then centrifuged for 8 minutes at 3100 rcf. After completion of all loading cycles, PDMS was poured onto the molds and cured as described for the elastomer-only pillars, incorporating the NPs into the resultant structure. Preliminary experiments showed that after 8 loading cycles, NPs overflowed the cylindrical holes of the pillar molds. Targeting pillars in which the upper half is loaded with NPs, four cycles of loading were used to prepare the arrays throughout this report.

### 2.4 Magnetic Field Application

To calibrate the NP-loaded pillars, a magnetic field was applied tangential to the array (horizontal in **Figure 1A** and perpendicular to individual pillars) by placing a spherical permanent earth magnet (SXO, 1” D spherical, N42, K&J magnetics) approximately 8 mm from the edge of the magnet to the edge of the pillar array. This allowed for an application of a 0.4 T field onto the pillars. To modulate the apparent spring constant of the pillars, a magnetic field was applied perpendicular to the array (vertical in **Figure 1A** and along the axis of individual pillars) using a cylindrical permanent magnet (DXO8B, N52, K&J magnetics) was mounted 7 mm above the pillar array, producing a field of 0.3 T at the pillar array surface. Magnetic field strength at the array position was confirmed using a gaussmeter (PCE-MFM 3500, PCE Instruments).

This report makes the simplifying assumption that magnetic field are uniform across the area of cell culture. To approximate a uniform magnetic field, the arrays were placed in line with the axis of the cylindrical magnet; it is recognized that the field produced by an individual, simple magnet is not completely uniform. However, the forces associated with such fields are small compared to those exerted by cells. In particular, the magnetic field along the axis of the DXO8B magnet was estimated to produce a gradient of approximately 300 Gauss/mm. For a coaxially oriented pillar with magnetic moment as described in the following section, this corresponds to a force of 1 pN, several orders of magnitude lower than those associated with cell traction forces.

### 2.5 Estimation of Pillar Mechanics

PDMS micropillars (not loaded with NPs) were modeled using Euler-Bernoulli beam theory (12) applied to a cylindrical beam of specified diameter (d), length (L), and material Young’s modulus (E); the spring constant (k) of this structure under a bending force (F) applied to the pillar tip and producing a resultant displacement (δ) can be estimated for small deflections as

$${\text{k}}_{\text{PDMS}}=\frac{\text{F}}{\delta }=\frac{3\pi \text{E}{\text{d}}^{4}}{64{\text{L}}^{3}}$$

**Figure 2:**
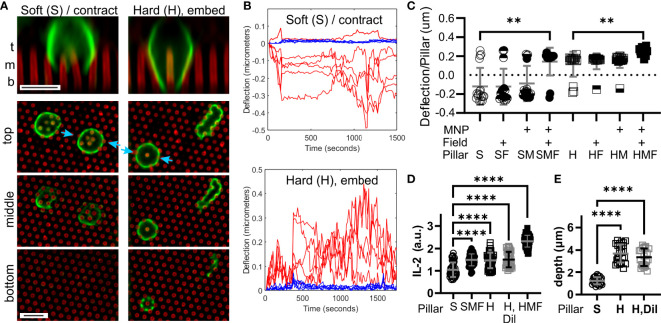
T cells respond to the microscale rigidity of micropillar arrays. **(A)** Comparison of mouse naïve CD4^+^ T cell interaction with arrays made of Soft (S) or Hard (H) PDMS. Focal planes representing the top, middle, and bottom of the arrays are shown, along with vertical projections collected at sites indicated by blue arrows. Cells were fixed 25 minutes after seeding, and were labeled with an anti-CD45 antibody (green). Pillars are shown in red. Scale bars = 5 µm. **(B)** Example trajectories illustrating deflections associated with a single cell on either Soft (S) or Hard (H) arrays. Red traces report deflections of individual pillars being manipulated by the cell, while blue traces illustrate movement of background pillars. These traces were derived from **Movies S1**, **S2**. Deflections towards the edge of the cell were assigned a positive sign, while those towards the cell center were negative. **(C)** Comparison of pillar deflections as a function of array formulation and magnetic field manipulation. Each data point reports the average deflection of all pillars underlying one cell. Data were compared using Kruskal-Wallis test, and only comparisons within a PDMS formulation are indicated; additional comparisons are discussed in the main text. Bars represent mean ± s.d., representing at least 15 cells per condition from 4 independent experiments. **(D)** Comparison of IL-2 secretion. Each data point represents one cell. Bars are mean ± s.d., representing at least 35 cells per condition from 3 independent experiments. **(E)** Cell penetration depth into the pillar arrays, measured by microscopy of cell morphology. Each data point represent one cell. Bars are mean ± s.d., representing at least 22 cells per condition from 3 independent experiments. Data were analyzed by ANOVA. For all analyses, **P < 0.01, ****P < 0.0001.

It is noted that the actual pillars exhibit a slight tapering, being narrower at the tip that interacts with the cells and wider at the base (**Figure 2A**). This tapering was incorporated into the fabrication process to allow better release of pillars from the molds. From images of fluorescently-labeled pillars (such as **Figure 2A**), pillars measured 1 µm diameter at a height of 2 µm from their base. It is noted that the same molds were used for all pillar formulations, so an adjustment to the spring constant calculation that would result from this slight tapering would affect all conditions equally. For this reason, all calculations were based on the design parameters of uniform, 1-µm diameter cross section, 6-µm height pillars.

Magnetic pillars were modeled as containing NPs in the upper half of each pillar, as suggested by the position of arrows indicating induced moments in **Figure 1A**. As an extreme approximation, it was considered that the NP-loaded material of each pillar is much stiffer than the non-loaded counterpart, E_PDMS+NP_ >> E_PDMS_. The spring constant of these structures in the absence of magnetic field was estimated using Castigliano’s method (19),

$${\text{k}}_{\text{PDMS}+\text{NP}}=\frac{3\pi \text{E}{\text{d}}^{4}}{64\left(L^{3}-{\left(\frac{L}{2}\right)}^{3}\right)}=\frac{3\pi \text{E}{\text{d}}^{4}}{56{\text{L}}^{3}}$$

The impact of an applied magnetic field on NP-loaded pillars was estimated by assuming a uniform magnetic field (B) that is along the axis of an individual pillar (B_mod_ in **Figure 1A**, perpendicular to the array). In this configuration, the induced magnetic field is aligned with the applied field, producing no torque (and under the assumption of a uniform, non-diverging field, no force). An applied force (F_cell_ in **Figure 1A**) will induce a misalignment between the pillar and field, resulting in a torque, τ = µ X B where µ is the pillar magnetic moment. In terms of magnitude, |τ| = |µ|*|B|*sin(θ) where θ is the angle between the µ and B vectors. For small displacements, θ ~ δ/L and the resultant τ would be associated with a restoring force F_mag_ = τ/L. Consequently, misalignment between µ and B results in a force F_mag_ that is proportional to the displacement δ, or

$${\text{k}}_{\text{mag}}=\frac{\text{F}}{\delta }=\frac{\mu \text{B}}{L^{2}}$$

The total apparent spring constant of a magnetically actuated pillar is thus the sum of k_PDMS+NP_ and k_mag_.

Finally, the induced moment of NP-loaded PDMS pillars in the presence of an applied magnetic field was estimated using the framework presented by Sniadecki et al. (19). In this case, a magnetic field (B, corresponding to B_calib_ in **Figure 1B**) is applied tangential to the array (perpendicular to an individual pillar) as specified for the configuration of Sniadecki et al. This configuration induces a deflection of a magnetically loaded pillar,

$$\delta =\frac{28{\text{L}}^{2}\tau }{\text{E}\pi {\text{d}}^{4}}∼\frac{28{\text{L}}^{2}\mu \text{B}}{\text{E}\pi {\text{d}}^{4}},$$

with the last approximation arising since in this configuration, sin(θ)~ 1.

### 2.6 Substrate Preparation

Pillars were coated with fluorescently labeled streptavidin (AlexaFluor 568, Thermo Fisher) at a concentration of 20 ug/mL, and then coated with biotinylated molecules anti-CD3 and anti-CD28 antibodies (eBioscience, clones 145-2C11 and 37.51) at a concentration of 20 ug/mL each, as described in previously (5). For experiments with diluted antibodies, anti-CD3 and anti-C28 were diluted with biotinylated rat anti-human IgG Antibody (BioLegend, 410718). Each step was performed for 1 hour at room temperature followed by 3X wash with PBS. The coated pillars were then immersed in complete media before cell seeding and imaging.

### 2.7 Immunostaining

Immunostaining was carried out using standard techniques. To label cell membranes, cells were stained with antibodies targeting CD45.2 (AlexaFluor 488, Biolegend, clone 104) before seeding onto substrates. For fixed-cell experiments, cells were fixed at the specified time points with 4% paraformaldehyde for 15 minutes, then washed 2X with PBS. To assay TCR activation, cells were stained with a primary antibody against phosphorylated Zap70 Tyr-319 (Biolegend B282374). The pZap70 signal was measured by fluorescence microscopy on cell-by-cell basis. All samples to be compared were included in each experiment, and all were stained, imaged, and processed in the same session. For experiments where a magnetic field was applied, the magnetic field was applied throughout the total incubation period upon cell seeding.

### 2.8 Cytokine Assays

Assays of IL-2 secretion were carried out using a surface-capture method as previously described (20, 21). Briefly, cells were incubated with an IL-2 capture reagent from a secretion assay kit (Miltenyi Biotec) before seeding. One hour after seeding, samples were rinsed with warm (37° C) RPMI-1640 media. After 6 hours of incubation (37°C), cells were rinsed and incubated on ice with a fluorescently labeled antibody to IL-2. The fluorescence intensity associated with APC-labeled IL-2 was measured by fluorescence microscopy on cell-by-cell basis. All samples to be compared were included in each experiment, and all were stained, imaged, background-subtracted, and processed in the same session to allow comparison among samples. For experiments where a magnetic field was applied, the magnetic field was applied throughout the total incubation period upon cell seeding.

### 2.9 Data Acquisition

Images were collected using an Olympus IX-81 fluorescence microscope with an Andor iXon3 EM-CCD and equipped with a 100X/1.45 NA Plan Apochromat objective (Olympus). Illumination channels 488, 568, and 647 nm were used for visualization of lymphocytes, pillars, cytokine markers, and fluorescent proteins. MetaMorph for Olympus was used to collect images. Image processing was performed with ImageJ/Fiji. Cell activity on pillars was carried out by seeding 1 x 10^5^ T cells in a 100 µl volume onto prepared pillars. Cell physical activity was recorded by live-cell microscopy in the half hour after seeding using a stage top incubator (Tokai). Images were collected at 15 second intervals over the 30-minute observation period. For experiments with magnetic field application, the custom magnetic rig was placed within the stage top incubator upon cell seeding.

Image processing was carried out using Fiji (22). Pillar displacements were tracked using the Particle Tracker plug-in (Mosaic ETH). Traces were then imported into MATLAB where background pillars were identified and cellular deflection measurements of pillars of interest were calculated. To determine the sign of a pillar deflection, pillars under the cell periphery were used to identify the cell centroid, which was denoted the center. Then, the sign of each pillar deflection was determined by calculating the dot product between the pillar displacement and vector from cell center to the specific pillar. Pillars that deflected away from the cell center were thus assigned a positive sign, while those deflected towards the cell center were given a negative sign. The average directionality was taken across the pillars under a single cell. Deconvolution of image stacks was carried out using the Deconvolution Lab plugin (23).

### 2.10 Statistical Analysis

Quantitative comparisons conducted using t-tests (for two conditions) and one way ANOVA (three or more conditions) for parametric data. Multiple comparisons, when justified by ANOVA, were carried out using Tukey’s range test methods. Kruskal-Wallis with Dunn’s multiple correction tests and Mann-Whitney tests were used for non-parametric data, as indicated for each analysis. Statistical tests were carried out using a significance level α = 0.05. All data is representative of at least two independent experimental runs, each of which contained multiple independent surfaces and discrete cell cultures.

### 2.11 Ethics Approval

The animal study was reviewed and approved by Columbia University’s Institutional Animal Care and Use Committee (IACUC).

## 3 Results

### 3.1 Fabrication and Characterization of Magnetic Pillar Arrays

The multi-micrometer-scale pillars often used for traction force microscopy are not suitable for use with T cells, given their comparatively small size (5 – 10 µm). As such, we adopt a standard geometry of 6-µm tall, 1-µm diameter pillars spaced in hexagonal arrays at 2 µm center-to-center spacing for this study. The small diameter of these pillars made high-occupancy loading of pillars using the nanowire approach (19) or others for fabricating larger, magnetically actuated cilia (24, 25) impractical. Instead, micro-scale magnetic structures were created by loading what will be the upper half of each elastomer with 10-nm superparamagnetic iron oxide nanoparticles (as detailed in *2.3 Magnetic Pillar Fabrication*); coupling between NPs in the presence of an applied field recapitulates the behavior of the nanowires, leading to torque generation (26). The ability of these nanoparticle-loaded pillars to respond to magnetic fields was measured using approaches developed by Sniadecki et al. (19). Specifically, a 0.4 T field placed tangential to the array (in the direction of B_calib_, **Figure 1B**) caused the tips of NP-loaded pillars cast of the standard Sylgard 184 elastomer (Young’s modulus, E, of 1.96 ± .09 MPa, mean ± s.d., n = 5, see *2.2 Elastomer Pillar Fabrication*) to deflect 1.98 ± 0.38 µm (mean ± s.d., n > 500 pillars); by comparison pillars that were not loaded with NPs showed negligible deflections. From this calibration, it was estimated (*2.5 Estimation of Pillar Mechanics*) that the application of a 0.3 T magnetic field perpendicular to the array (B_mod_ in **Figure 1A**) would impart a spring constant, k_mag_, of 0.38 nN/µm.

Combining this approach with modulation of PDMS formulation produced a series of pillars presenting a range of estimated spring constants shown in **Figure 1C**. When cast from the standard Sylgard 184 elastomer, denoted “Hard” PDMS, the spring constant of each pillar is estimated to be 1.34 nN/µm (H in **Figure 1C**). Loading the upper half of each pillar with NPs is estimated to increase its spring constant to 1.53 nN/µm (HM, section *2.5 Estimation of Pillar Mechanics*). This calculation is based on E_PDMS+NP_ >> E_PDMS_, but even with this extreme approximation, the pillar spring constant increased by only 14%. Application of the magnetic field B_mod_ (HMF in **Figure 1C**) is estimated to increase the pillar total spring constant to 1.91 nN/µm, an increase of 43% compared to the PDMS pillar. Pillars were also cast from a comparatively soft Sylgard 527 elastomer mixed 1: 3 (v/v) with Sylgard 184. This mixture, denoted “Soft”, exhibited a Young’s modulus of 1.01 ± .06 MPa (mean ± s.d., n = 5) yielding the pillars designated as S in **Figure 1C** along with the NP-loaded condition SM and magnetically actuated SMF. Notably, the estimated spring constant for SMF is similar to that of H, allowing comparison between pillars made of the different PDMS formulations.

### 3.2 T Cells Respond to Pillar Spring Constant

The ability T cells to recognize differences in macroscopic rigidity was tested using the micropillar array series defined in **Figure 1C**. Comparing the interaction of mouse naïve CD4^+^ T cells with micropillar arrays cast from the Sylgard 184 (“Hard”) and Sylgard 527 + Sylgard 184 mix (“Soft”) PDMS formulations revealed two distinct types of interaction. Within minutes of contact with S arrays, T cells exhibited a contractile morphology, deflecting groups of pillar towards the center of the cell – substrate interface (**Figures 2A, B** and **Movie S1**). Cells extended processes partly into the arrays, but did not reach the bottom of the pillars. In contrast, cells on the H arrays embedded deeply between pillars, extending processes to the bottom of the arrays and pushing pillars towards the edge of the cell (**Figures 2A, B** and **Movie S2**). A lone pillar embedded inside these cells was frequently observed (vertical projection in **Figure 2A** and indicated by blue arrows), which would often be manipulated at later timepoints in the 30 minute observation period. Towards a cell-level representation of these behaviors, deflections of pillars towards the cell edge were assigned a positive sign, while those towards the cell center were negative in magnitude. These signed deflections were averaged over all pillars under a cell and used as a single measure of cell state, with positive average deflections suggesting an embedding interaction and negative reflecting a contractile state. The propensity of cells to exhibit contraction and embedding on S and H pillar arrays respectively is seen in **Figure 2C** (P < 0.005, Mann Whitney test). Notably, the contractile and embedding cell morphologies were stable on each substrate up to 6 hours, the longest that was examined in this report.

Comparisons of cell response across the entire pillar series addressed whether the change in cell interaction between the S and H pillars was associated with spring constant (structural rigidity) or elastomer formulation (local stiffness). For the Soft series, application of the magnetic field switched T cells from contractile to embedding interaction (S vs. SMF, **Figure 2C**). This effect was not observed for pillars either loaded with NPs (SM) or subjected to a magnetic field (SF), indicating that neither modification alone provided this effect. Notably, cell response on the SMF arrays was similar to that on the H arrays (P > 0.99), indicating that pillar spring constant, rather than difference in formulation between Hard and Soft PDMS was associated with promoting an embedded rather than contractile morphology. Finally, application of a magnetic field to NP-loaded Hard arrays further increased average deflection (**Figure 2C**) indicating that this effect extended to higher spring constants.

To determine if pillar spring constant had a functional impact on T cell response, we next compared secretion of the cytokine IL-2. This was measured using a cell-surface capture assay over six hours, providing a cell-by-cell picture of activation. As shown in **Figure 2D**, the H pillars promoted a stronger IL-2 response than the S counterparts. In addition, SMF pillars exhibited IL-2 secretion that was higher than the S arrays, and similar to H, indicating that a higher spring constant induced stronger IL-2 secretion. IL-2 secretion was highest on HMF arrays (P < 0.001 comparison with H but not indicated on **Figure 2D**), supporting the observation that increasing spring constant produced higher activation. However, it is noted that the H arrays also promoted more interaction between cells and pillars than S (**Figure 2E**), and it is possible that simply being exposed to additional activating antibody on the pillar walls led to enhanced IL-2 secretion. To address this possibility, 46% of the activating antibodies in the coating solution was replaced with an inert counterpart, reflecting the difference in process extension depth between S and H surfaces (**Figure 2E**). This dilution had no effect on IL-2 secretion or process extension length (**Figures 2D, E**), indicating that simply access to activating antibody does not explain the observed differences.

**Figure 3:**
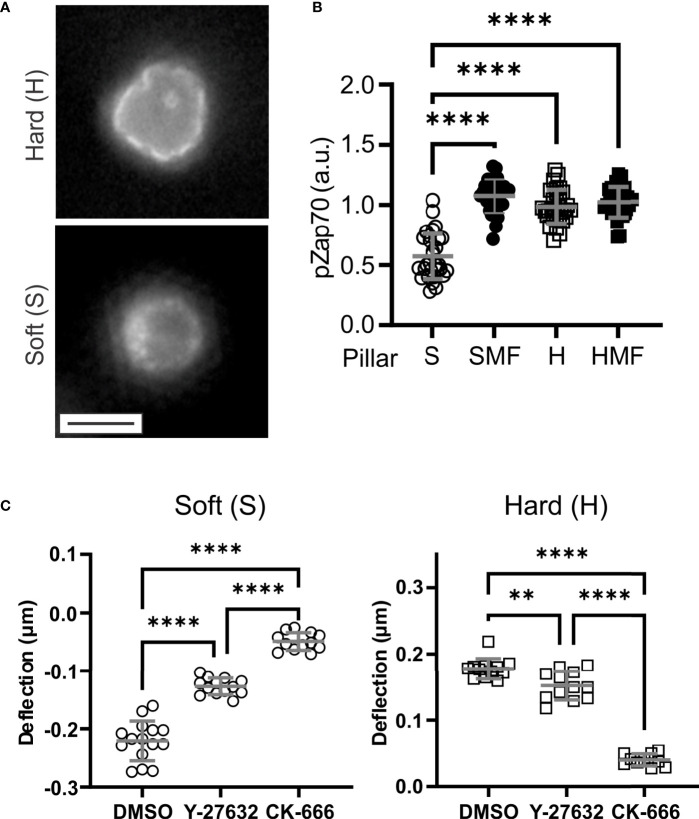
Molecular mechanisms of T cell mechanosensing. **(A)** Distribution of phsopho-Zap70 (Tyr 319). Cells were fixed 15 minutes after seeding. These images illustrate a plane through cells at the pillar tips. Scale bar = 5 µm. **(B)** Quantitative comparison of pZap70. Each data point represents an individual cell, and bars report mean ± s.d., from at least 30 cells per condition from 3 independent experiments. Only significant comparisons with the Soft (S) condition are indicated; additional comparison are reported in the main text. **(C)** Effect of inhibitors of cytoskeletal dynamics on pillar deflection. Each data point represents an individual cell, and bars report mean ± s.d., from at least 15 cells per condition from 3 independent experiments. DMSO = vehicle control for both inhibitors. For all panels, data were analyzed by one way ANOVA; **P < 0.01, ****P < 0.0001.

Towards molecular insights into T cell sensing of pillar rigidity, Zap70 activation was compared 15 minutes after seeding onto the substrates. On the soft S arrays, phospho-Zap70 (pZap70, Tyr319) was localized in small clusters along the cell membrane (**Figure 3A**). On H arrays, membrane localization was more uniform, conforming with the cell shape and even around pillars that protruded into the cells. Total pZap70 staining, measured on a cell-by-cell basis, was higher on more rigid SMF, H, and HMF conditions (**Figure 3B**), mirroring the wider localization suggests by microscopy. This result suggests that increased spring constant is associated with stronger TCR activation. However, no difference was detected between the HMF and SMF or H formulations. This was unexpected since HMF promoted higher IL-2 secretion than the other two systems, and suggests that additional aspects of cell signaling, such as duration of Zap70 phosphorylation or recruitment of alternative pathways, are involved in T cell mechanosensing sensing.

### 3.3 Actin Polymerization Contributes to T Cell Embedding

This final section explores the systems of cytoskeletal dynamics that are involved with the ability of T cells to conform to and manipulate the pillar arrays. This was carried out by pretreating T cells with inhibitors of actin dynamics before seeding on arrays. Inhibition of Apr2/3-mediated actin polymerization with CK-666 largely abrogated pillar deflection on both S and H surfaces (**Figure 3C**). The effect was more pronounced on the H surfaces, for which cells did not embed into the micropillar arrays; cells on the S surfaces remained on the pillar tops. Pretreatment with the Rho/ROCK inhibitor Y-27632 had a smaller effect on cells, reducing pillar deflections but not to the same extent as CK-666. In addition, cells on the H surfaces in the presence of Y-27632 were able to reach the bottom of the arrays. These results indicate a major role of actin polymerization in both contractile and embedding morphologies. We note that these results do not consider all pathways modulating actomyosin contractility in T cells, and indeed blebbistatin is often included as an inhibitor for studies of mechanosensing (5). However, the hydrophobic characteristic of blebbistatin makes its use in the presence of PDMS substrates, which absorb hydrophobic molecules, problematic; for this reason, inhibition of contractility through this mechanism was not explored in the present study.

## 4 Discussion

Forces play increasingly recognized roles in T cell activation at many levels of organization. At the molecular level, mechanical loading of the TCR – Major Histocompatibility Complex (MHC) alters bond lifetime revealing a catch-bond behavior for specific subsets of interactions (27–29). Complementary studies using substrate-immobilized anti-CD3 and anti-CD28 revealed a cell-wide role of forces, in which forces applied to CD3 leads to TCR activation and the ability of T cells to sense the mechanical stiffness of an underlying substrate (3, 5, 30–33); the TCR-pMHC catch binds is an additional layer of complexity over this CD3 mechanical response. However, studies of T cell mechanosensing, like those for other cell types (34, 35), rely predominantly on hydrogels (such as polyacrylamide (PA) or alginate) or PDMS elastomer. These two systems provide different capabilities and benefits for mechanosensing studies, but neither is without complications (16). Many of these considerations center around the concept that changing mechanical stiffness involves altering the concentration of components (such as crosslinkers) if not the formulation itself. The use of photoactive crosslinkers, bivalent ions, and nucleic acid chains to alter stiffness provide evidence that mechanosensing is independent of chemical formulation, but do not completely address this question. The use of an applied magnetic field to alter rigidity avoids the use of chemical and exposure to light, and promises reversible, rapid modulation. Our system is a first demonstration of this approach, seen most directly in the difference in response between SM and SMF arrays (**Figure 2C**). This approach also avoids considerations that changing the pillar dimensions many have on cell response (10, 13, 15); we anticipate that the system presented here can be used for other cell types, including fibroblasts, epithelial cells, and stem cells.

Magnetic manipulation of pillar spring constant along with changing PDMS formulation also demonstrated that T cells are sensitive to structural rigidity; while nanoscale, molecular effects such as catch bonds influence signaling, the mechanical resistance of a system at the scale of micrometers can be recognized by these cells. The S configuration represents low material stiffness and structural rigidity, promoting a contractile response from cells. Cytoskeletal inhibition assays suggest that branching actin polymerization contributes to this morphology, potentially by promoting centripetal flow of material from the cell edge towards the center. Inhibition of actomyosin contractility (through the Rho/ROCK inhibitor Y-27632) also reduced inward pillar deflection, supporting the concept that these systems interact in defining the cytoskeletal state of T cells. Increasing pillar spring constant by either magnetic manipulation (SMF) or using a stiffer PDMS (H) increased cell activation as evidenced by increased IL-2 secretion. This was accompanied by a switch to the embedding morphology, which resembles amoeboid migration of T cells which is dependent on robust polymerization of actin through Arp2/3. Such polymerization would underlie the observed extension of processes between pillars and deflection of pillars away from the cell center as supported by experiments of CK-666 inhibition on the H arrays. It is recognized that T cell activation is a complex process, and full understanding of how nanoscale, molecular mechanosensing and microscale structural rigidity influence each other remains incomplete. Further studies using the tools introduced here could shed new light into signaling withing the complex T cell – APC interface, as well as provide new strategies for designing biomaterials that modulate adaptive immunity.

## Data Availability Statement

The raw data supporting the conclusions of this article will be made available by the authors, without undue reservation.

## Ethics Statement

The animal study was reviewed and approved by Institutional Animal Care and Use Committee, Columbia University.

## Author Contributions

CS and LK designed the study and also carried out data collection and analysis. CS conducted the experiments. All authors contributed to the article and approved the submitted version.

## Funding

This work was supported in part by the National Institutes of Health (R21AI119953 and R01AI110593 to LK).

## Conflict of Interest

The authors declare that the research was conducted in the absence of any commercial or financial relationships that could be construed as a potential conflict of interest.

## Publisher’s Note

All claims expressed in this article are solely those of the authors and do not necessarily represent those of their affiliated organizations, or those of the publisher, the editors and the reviewers. Any product that may be evaluated in this article, or claim that may be made by its manufacturer, is not guaranteed or endorsed by the publisher.

## Supplementary Material

Supplementary Movie S1 Manipulation of pillars by a cell exhibiting a contractile interaction. This video shows motion of pillar tops being manipulated by a cell on a Soft (S) array. Timestamp is in MM : SS, and scale bar = 5 µm.

Supplementary Movie S2 Manipulation of pillars by a cell exhibiting an embeddinginteraction. This video shows motion of pillar tops being manipulated by a cell on a Hard (H) array. Timestamp is in MM : SS, and scale bar = 5 µm.
